# Supplementary material for: Dimensionality Matters: Exploiting UV-Photopatterned 2D and Two-Photon-Printed 2.5D Contact Guidance Cues to Control Corneal Fibroblast Behavior and Collagen Deposition
Source: Bioengineering (Basel). 2024 Apr 19;11(4):402. doi: 10.3390/bioengineering11040402 (PMC11048187; doi:10.3390/bioengineering11040402)
Supplement: Supplementary file 1 [file bioengineering-11-00402-s001.zip › bioengineering-2972625-supplementary.pdf]

Article

Dimensionality Matters: Exploiting UV-Photopatterned 2D and Two-Photon-Printed 2.5D Contact Guidance Cues to Control Corneal Fibroblast Behavior and Collagen Deposition

Cas van der Putten [1,2,†], Gozde Sahin [3,†], Rhiannon Grant [3], Mirko D'Urso [1,2], Stefan Giselbrecht [3], Carlijn V. C. Bouten [1,2] and Nicholas A. Kurniawan [1,2,*]

[1] Department of Biomedical Engineering, Eindhoven University of Technology, P.O. Box 513, 5600 MB Eindhoven, The Netherlands; casvanderputten@gmail.com (C.v.d.P.); m.d.urso@tue.nl (M.D.); c.v.c.bouten@tue.nl (C.V.C.B.)
[2] Institute for Complex Molecular Systems, Eindhoven University of Technology, P.O. Box 513, 5600 MB Eindhoven, The Netherlands
[3] MERLN Institute for Technology-Inspired Regenerative Medicine, Department of Cell Biology-Inspired Tissue Engineering cBITE, 6229 ER Maastricht, The Netherlands; g.sahin@maastrichtuniversity.nl (G.S.); rhiresearch@proton.me (R.G.); s.giselbrecht@maastrichtuniversity.nl (S.G.)
* Correspondence: n.a.kurniawan@tue.nl
† These authors contributed equally to this work.

Supplementary Information

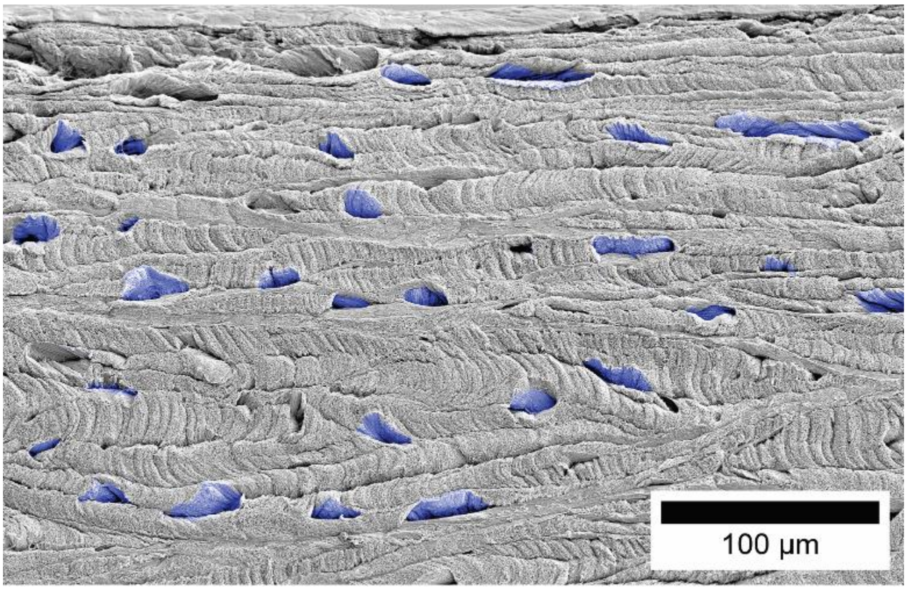

Figure S1. False colored SEM image of the corneal stroma cross-section shows voids in blue, which are possible hosts where keratocytes reside in their native environment.

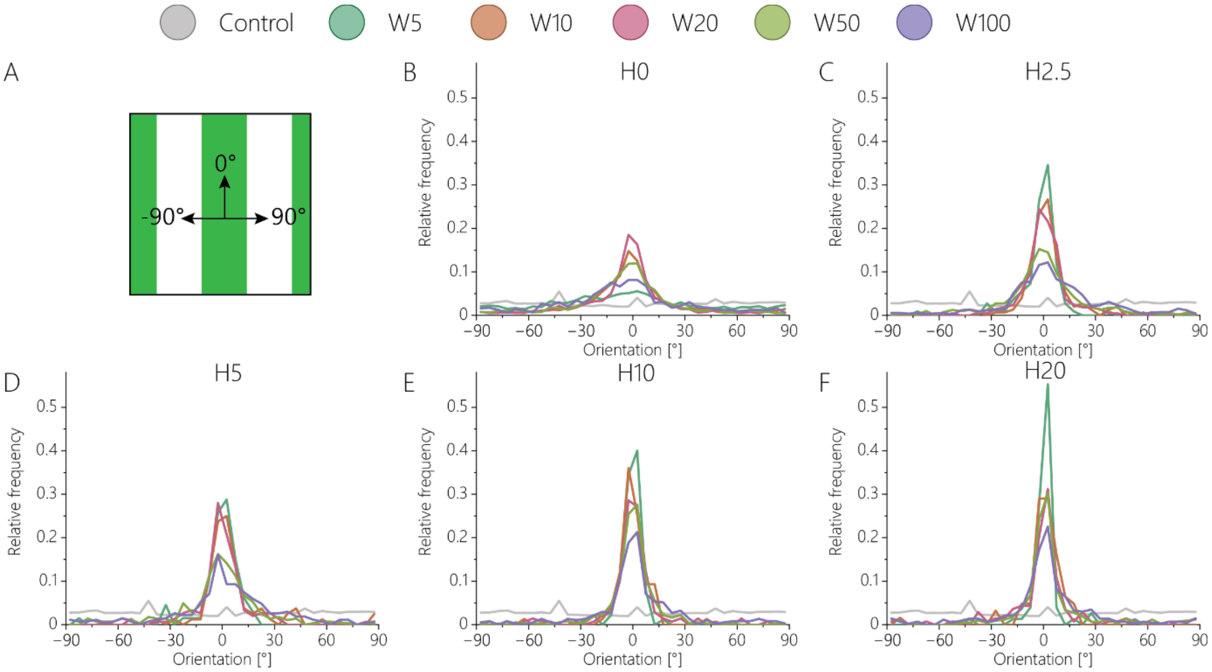

Figure S2. The orientation distributions of activated human primary keratocytes on contact guidance cue presenting substrates. A) Quantification of orientation with respect to the cue direction, which means 0° represents cell alignment towards pattern line direction. Orientation distributions of B) 2D cues (H0), 2.5D cues of C) 2.5 μm, D) 5 μm, E) 10 μm, and F) 20 μm

tall; -90° represents a counter-clockwise rotation, and 90° represents a clockwise rotation. Statistical differences between distributions are described in Table S1.

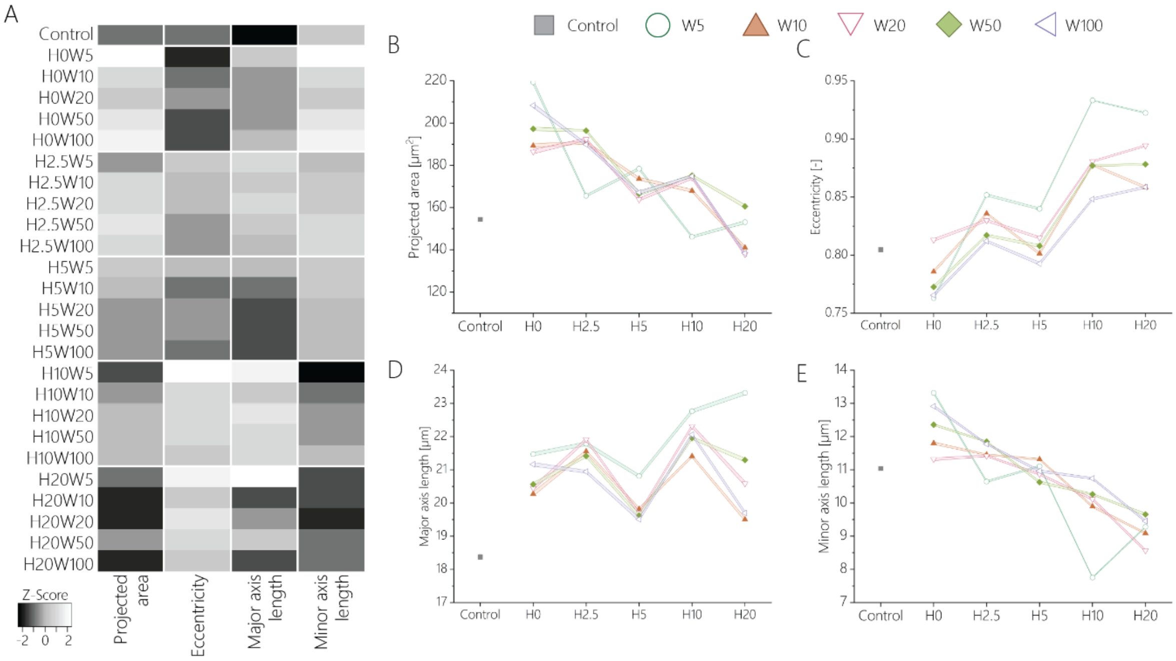

Figure S3. A) Heatmap of morphological parameters of the nuclei of fibroblastic keratocytes when cultured on substrates presenting 2.5D and 2D contact guidance cues. Gray scales are normalized for each readout. B) The projected area, C) eccentricity, D) major axis length, and E) minor axis length of activated keratocytes on substrates with and without (control, grey) cues. All data are presented as mean ± standard error of the mean. Statistical differences between conditions are described in Tables S2-S5.

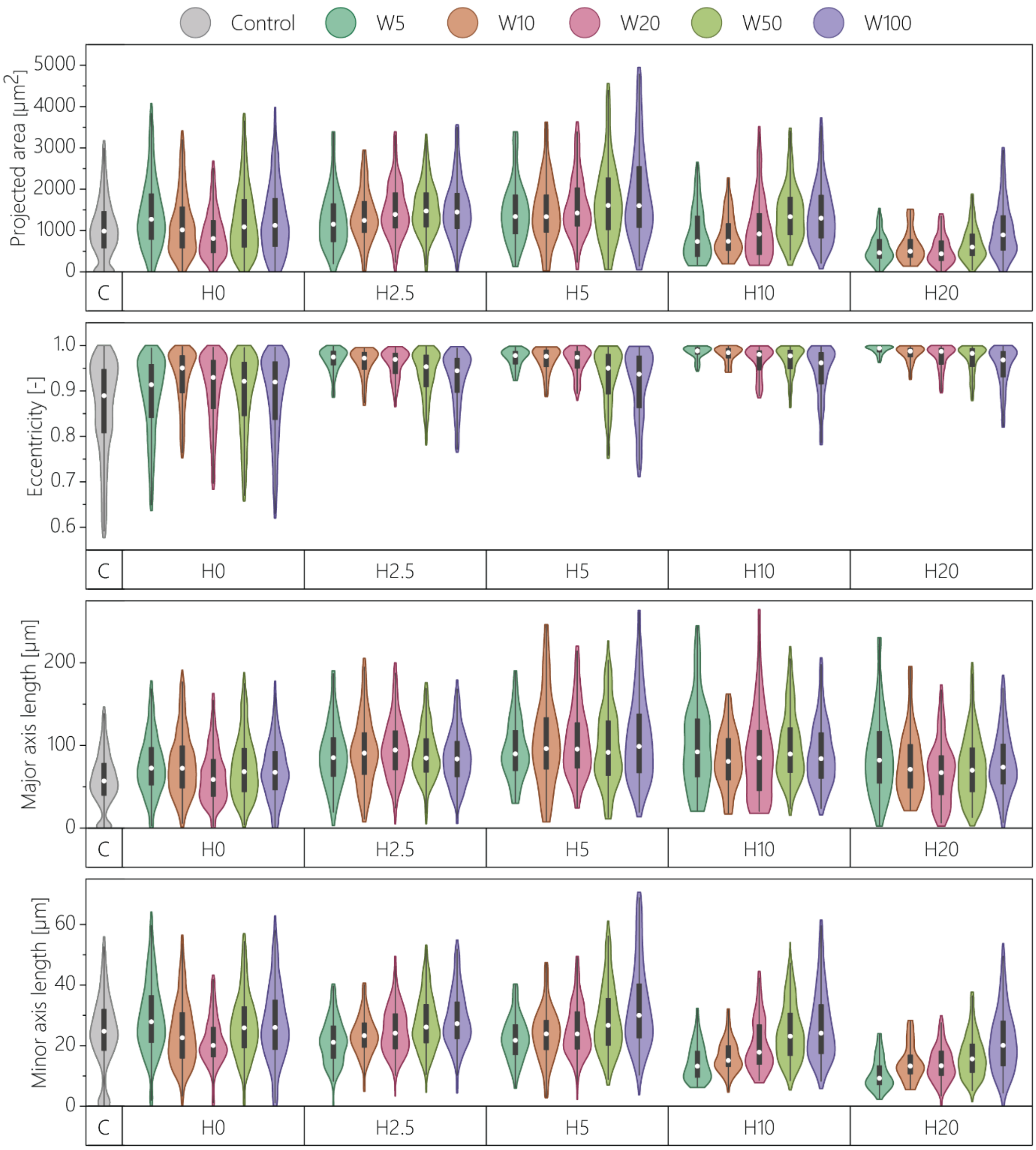

Figure S4. Violin plots for the projected area, eccentricity, major and minor axis length of fibroblastic keratocytes on contact guidance cue presenting substrates. The white points represent the median of each violin dataset. Statistical differences between conditions are described in Tables S2-S5.

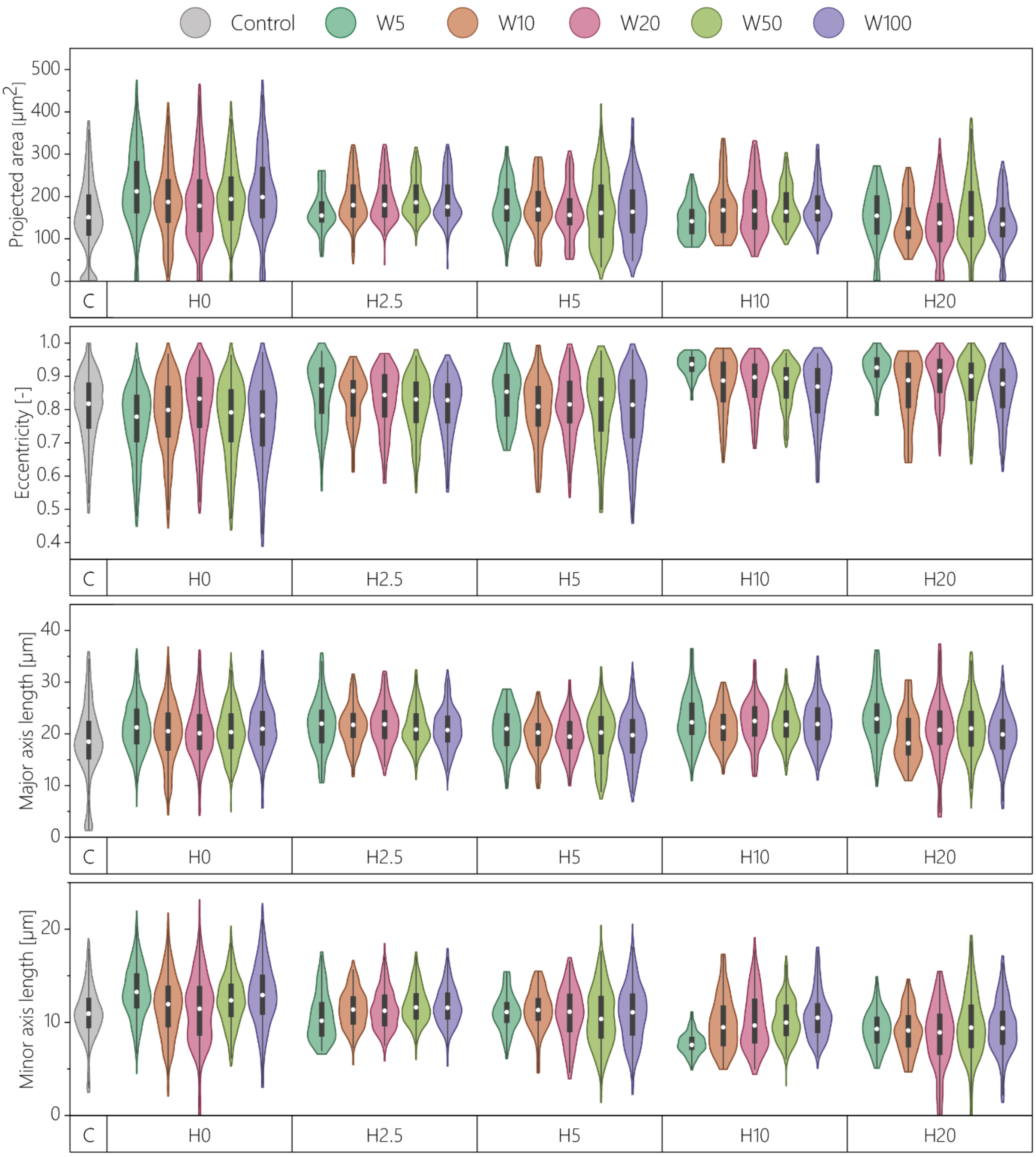

Figure S5. Violin plots for the projected area, eccentricity, major and minor axis length of nuclei on contact guidance cue presenting substrates. The white points represent the median of each violin dataset. Statistical differences between conditions are described in Tables S2-S5.

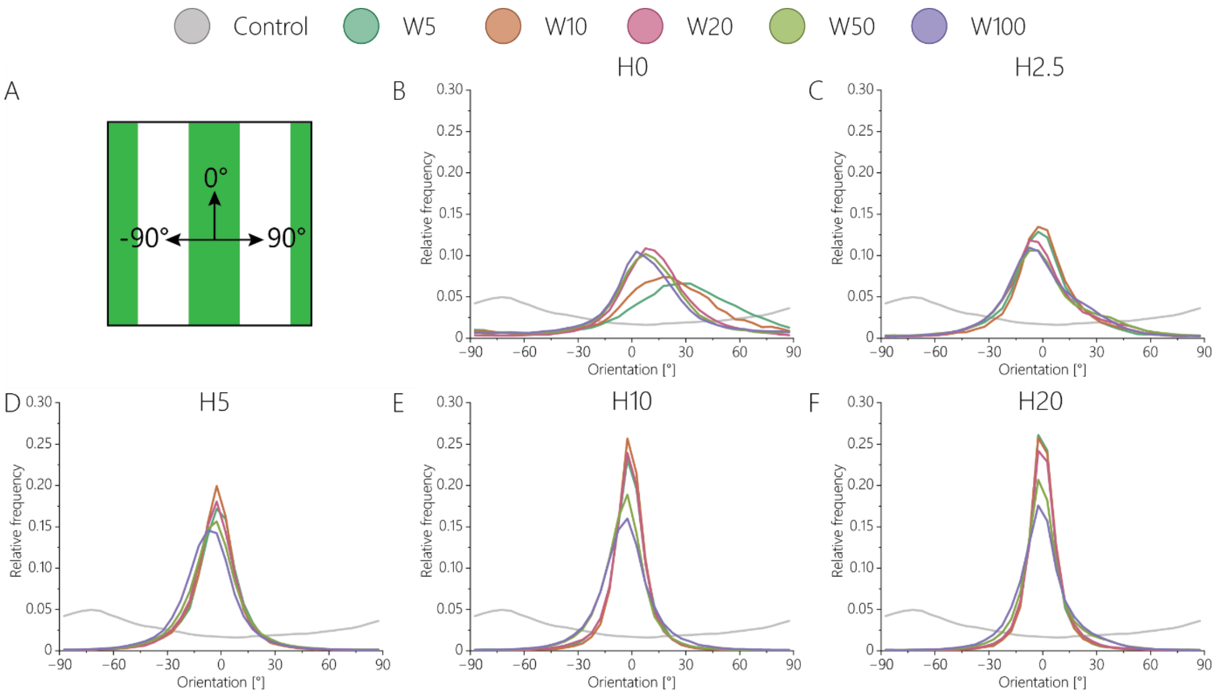

Figure S6. The orientation distributions of fibroblastic keratocytes with respect to the contact guidance cues, meaning that 0° represents cells aligning according to the protein pattern (H0) or 2.5D cues (H2.5-H20) (A). Orientation distributions of B) H0, C) H2.5, D) H5, E) H10, and F) H20; -90° represents a counter-clockwise rotation, and 90° represents a clockwise rotation.

Table S1. Statistical analysis for the orientation distributions of activated keratocytes (red) and nuclei (blue) for all conditions with respect to each other. – Not significant, * $p<0.05$, ** $p<0.01$, *** $p<0.001$, **** $p<0.0001$.

H20W100	****	****	****	****	****	****	***	–	*	**	***	*	–	*	*	*	****	*	**	**	–	***	–	*	–	
H20W50	****	****	****	****	****	****	–	–	–	****	****	–	–	–	***	****	**	–	–	–	–	**	–	–		*
H20W20	****	****	****	***	****	****	–	–	–	**	****	–	–	–	**	***	–	–	–	–	–	*	–		–	**
H20W10	****	****	***	***	****	****	–	–	–	**	***	–	–	–	**	***	–	–	–	–	–	*		–	–	–
H20W5	****	****	****	****	****	****	*	**	**	****	****	*	**	**	****	****	–	*	*	**	***		**	–	*	***
H10W100	****	****	****	***	****	****	***	–	*	*	***	*	*	–	–	**	****	*	*	**		**	–	*	–	–
H10W50	****	****	****	****	****	****	–	–	–	***	****	–	–	–	**	****	**	–	–		–	*	–	–	–	*
H10W20	****	****	****	***	****	****	–	–	–	**	****	–	–	–	*	****	–	–		–	*	*	–	–	–	–
H10W10	****	****	****	**	****	****	–	–	–	**	****	–	*	–	*	****	–		–	–	–	–	–	–	–	–
H10W5	****	****	****	***	****	****	–	**	***	****	****	–	***	*	****	****		*	**	***	****	–	***	*	****	****
H5W100	****	****	***	****	****	****	****	***	****	**	–	****	*	****	**		****	***	***	****	*	****	–	****	****	*
H5W50	****	****	–	–	**	****	**	**	*	–	***	–	**	–		–	****	*	**	**	–	****	–	***	**	–
H5W20	****	****	****	**	****	****	–	–	–	*	****	–	*		–	**	***	–	*	–	–	**	–	–	*	–
H5W10	****	****	****	****	****	****	*	–	*	***	**	–		–	–	–	****	*	*	–	–	***	–	**	*	–
H5W5	****	****	**	–	***	****	–	–	–	*	****		–	–	–	–	****	–	–	–	–	**	–	*	–	–
H2.5W100	****	****	****	****	****	****	****	****	****	***		–	–	***	–	–	****	***	****	****	**	****	*	****	****	***
H2.5W50	****	****	–	*	**	****	***	***	**		**	*	*	–	–	–	****	*	****	****	*	***	–	**	****	***
H2.5W20	****	****	****	***	****	****	*	–		–	****	–	–	–	*	***	***	–	**	*	–	**	–	–	**	*
H2.5W10	****	****	****	****	****	****	–		–	*	–	–	–	–	–	–	****	*	–	*	–	***	–	**	–	–
H2.5W5	****	****	***	**	****	****		–	–	**	***	–	–	–	*	**	**	–	–	–	–	–	–	–	–	–
H0W100	****	***	****	****	***		****	****	****	****	****	****	****	****	****	****	****	****	****	****	****	****	****	****	****	****
H0W50	****	****	–	****		***	***	****	**	*	***	**	**	**	**	***	****	***	****	****	****	****	**	***	****	****
H0W20	****	****	**		****	****	*	*	–	–	****	–	*	–	–	***	****	*	****	****	*	***	–	*	****	****
H0W10	****	****		****	–	****	**	**	**	–	***	**	**	*	–	*	****	**	****	****	****	****	*	**	****	****
H0W5	****		****	****	****	–	****	****	****	****	****	****	****	****	****	****	****	****	****	****	****	****	****	****	****	****
C		****	****	****	****	****	****	****	****	****	****	****	****	****	****	****	****	****	****	****	****	****	****	****	****	****
	C	H0W5	H0W10	H0W20	H0W50	H0W100	H2.5W5	H2.5W10	H2.5W20	H2.5W50	H2.5W100	H5W5	H5W10	H5W20	H5W50	H5W100	H10W5	H10W10	H10W20	H10W50	H10W100	H20W5	H20W10	H20W20	H20W50	H20W100

Table S2. Statistical analysis for the projected area of activated keratocytes (red) and nuclei (blue) for all conditions with respect to each other. – Not significant, * p<0.05, ** p<0.01, *** p<0.001, **** p<0.0001.

	C	H0W5	H0W10	H0W20	H0W50	H0W100	H2.5W5	H2.5W10	H2.5W20	H2.5W50	H2.5W100	H5W5	H5W10	H5W20	H5W50	H5W100	H10W5	H10W10	H10W20	H10W50	H10W100	H20W5	H20W10	H20W20	H20W50	H20W100
H20W100	–	****	**	*	****	****	***	****	****	****	****	****	****	****	****	****	–	–	–	****	****	****	****	****	****	
H20W50	****	****	****	****	****	****	****	****	****	****	****	****	****	****	****	****	*	**	****	****	****	*	–	***		**
H20W20	****	****	****	****	****	****	****	****	****	****	****	****	****	****	****	****	****	****	****	****	****	–	–		*	–
H20W10	****	****	****	****	****	****	****	****	****	****	****	****	****	****	****	****	*	**	***	****	****	–		–	–	–
H20W5	****	****	****	****	****	****	****	****	****	****	****	****	****	****	****	****	**	****	****	****	****		–	–	–	*
H10W100	****	–	****	****	***	***	–	–	*	***	**	–	–	**	****	****	****	****	****	–		*	****	****	****	****
H10W50	****	–	****	****	****	***	*	–	–	**	**	–	–	*	****	****	****	****	****		–	*	****	****	****	****
H10W20	–	****	–	–	**	**	**	****	****	****	****	***	***	****	****	****	–	–		–	–	–	***	****	*	****
H10W10	*	****	**	–	***	***	****	****	****	****	****	****	****	****	****	****	–		–	–	–	–	*	**	–	**
H10W5	*	****	**	–	****	****	****	****	****	****	****	****	****	****	****	****		*	**	****	****	–	–	–	–	–
H5W100	****	****	****	****	****	****	****	****	**	**	**	**	*	–	–		*	–	–	–	–	–	**	***	–	****
H5W50	****	****	****	****	****	****	****	***	*	–	*	*	–	–		–	–	–	–	*	*	–	*	**	–	****
H5W20	****	**	****	****	****	****	***	*	–	–	–	–	–		–	–	*	–	–	–	–	–	**	**	–	****
H5W10	****	–	***	****	**	*	–	–	–	–	–	–		–	–	–	***	–	–	–	–	–	***	***	*	****
H5W5	****	–	***	****	*	*	–	–	–	–	–		–	–	–	–	***	–	–	–	–	–	***	****	*	****
H2.5W100	****	****	****	****	****	****	***	**	–	–		–	–	****	****	****	****	***	**	****	****	****	****	****	****	****
H2.5W50	****	****	****	****	****	****	****	**	–		*	**	**	****	****	****	****	****	****	****	****	****	****	****	****	****
H2.5W20	****	**	****	****	****	****	***	*		–	–	–	–	****	****	****	****	***	**	***	***	****	****	****	****	****
H2.5W10	****	–	****	****	**	*	–		–	–	–	–	–	****	***	***	****	**	**	***	***	***	****	****	****	****
H2.5W5	**	–	*	****	–	–		***	****	****	****	–	–	–	–	–	**	–	–	–	–	–	***	***	–	****
H0W100	****	****	**	****	–		****	*	*	–	***	**	**	****	****	****	****	****	****	****	****	****	****	****	****	****
H0W50	****	****	**	****		*	****	–	–	–	–	–	*	****	****	****	****	***	***	****	****	****	****	****	****	****
H0W20	****	****	****		****	****	–	–	*	****	*	–	–	*	***	**	****	–	–	–	–	*	****	****	****	****
H0W10	**	****		*	*	****	***	–	–	–	–	–	–	****	****	****	****	**	*	***	****	***	****	****	****	****
H0W5	****		****	****	****	**	****	****	****	****	****	****	****	****	****	****	****	****	****	****	****	****	****	****	****	****
C		****	****	****	****	****	–	****	****	****	****	**	*	–	–	*	–	–	*	****	****	–	–	*	–	****

Table S3. Statistical analysis for the eccentricity of activated keratocytes (red) and nuclei (blue) for all conditions with respect to each other. – Not significant, * p<0.05, ** p<0.01, *** p<0.001, **** p<0.0001.

	C	H0W5	H0W10	H0W20	H0W50	H0W100	H2.5W5	H2.5W10	H2.5W20	H2.5W50	H2.5W100	H5W5	H5W10	H5W20	H5W50	H5W100	H10W5	H10W10	H10W20	H10W50	H10W100	H20W5	H20W10	H20W20	H20W50	H20W100
H20W100	****	****	****	****	****	****	*	-	-	****	****	*	*	-	****	****	****	****	*	***	*	****	****	****	****	
H20W50	****	****	****	****	****	****	*	****	****	****	****	-	-	**	****	****	***	-	-	*	****	****	*	-		***
H20W20	****	****	****	****	****	****	**	****	****	****	****	*	*	***	****	****	*	-	*	**	****	****	-		*	****
H20W10	****	****	****	****	****	****	***	****	****	****	****	**	**	****	****	****	-	-	**	***	****	**		*	-	-
H20W5	****	****	****	****	****	****	****	****	****	****	****	****	****	****	****	****	**	****	****	****	****		***	*	****	****
H10W100	****	****	***	****	****	****	***	*	*	-	****	***	**	**	*	****	****	****	****	****		****	-	****	****	-
H10W50	****	****	****	****	****	****	-	*	***	****	****	-	-	-	****	****	****	**	-		***	****	-	***	-	**
H10W20	****	****	****	****	****	****	-	-	**	****	****	-	-	-	****	****	****	**		-	***	***	-	-	-	**
H10W10	****	****	****	****	****	****	**	****	****	****	****	**	**	***	****	****	-		-	-	**	**	-	-	-	*
H10W5	****	****	****	****	****	****	****	****	****	****	****	****	****	****	****	****		****	****	****	****	-	****	***	****	****
H5W100	****	****	*	*	***	****	****	****	****	**	-	****	****	****	*		****	****	****	****	****	****	****	****	****	****
H5W50	****	****	-	****	****	****	****	****	****	-	-	****	****	****		-	****	****	****	****	****	****	***	****	****	****
H5W20	****	****	****	****	****	****	-	-	-	****	****	-	-		-	-	****	****	****	****	****	****	**	****	****	****
H5W10	****	****	****	****	****	****	-	-	*	****	****	-		-	-	-	****	****	****	****	****	****	***	****	****	****
H5W5	****	****	****	****	****	****	-	-	*	****	****		*	-	-	*	****	**	***	***	-	****	-	***	***	*
H2.5W100	****	****	*	****	****	****	****	****	****	**		*	-	-	-	-	****	****	****	****	****	****	****	****	****	****
H2.5W50	****	****	-	****	****	****	****	****	****		-	-	-	-	-	-	****	****	****	****	****	****	***	****	****	****
H2.5W20	****	****	****	****	****	****	*	-		*	**	-	*	-	-	**	****	****	****	****	**	****	*	****	****	***
H2.5W10	****	****	****	****	****	****	-		-	*	**	-	**	-	-	**	****	****	****	****	*	****	*	****	****	***
H2.5W5	****	****	****	****	****	****		*	*	****	****	-	***	**	***	****	****	*	*	-	-	****	-	***	**	-
H0W100	****	-	****	***	-		****	****	****	****	****	****	*	****	****	**	****	****	****	****	****	****	****	****	****	****
H0W50	****	-	****	**		-	****	****	****	****	****	****	-	***	****	**	****	****	****	****	****	****	****	****	****	****
H0W20	****	****	****		****	****	***	-	-	-	-	-	-	-	-	**	****	****	****	****	****	****	***	****	****	****
H0W10	****	****		****	*	***	****	****	****	****	****	***	-	**	***	-	****	****	****	****	****	****	****	****	****	****
H0W5	****		****	****	**	-	****	****	****	****	****	****	**	****	****	****	****	****	****	****	****	****	****	****	****	****
C		****	****	****	****	****	****	***	***	-	-	*	-	-	-	-	****	****	****	****	****	****	****	****	****	****

Table S4. Statistical analysis for the major axis length of activated keratocytes (red) and nuclei (blue) for all conditions with respect to each other. – Not significant, * p<0.05, ** p<0.01, *** p<0.001, **** p<0.0001.

	C	H0W5	H0W10	H0W20	H0W50	H0W100	H2.5W5	H2.5W10	H2.5W20	H2.5W50	H2.5W100	H5W5	H5W10	H5W20	H5W50	H5W100	H10W5	H10W10	H10W20	H10W50	H10W100	H20W5	H20W10	H20W20	H20W50	H20W100
H20W100	****	-	-	****	**	**	**	***	****	****	***	***	****	****	****	****	***	-	-	****	***	-	-	**	*	
H20W50	****	*	*	****	-	-	***	****	****	****	****	****	****	****	****	****	****	*	**	****	****	*	-	-		****
H20W20	*	**	**	-	-	-	****	****	****	****	****	****	****	****	****	****	****	**	**	****	****	**	-		-	-
H20W10	**	-	-	**	-	-	*	*	***	**	*	**	**	****	***	****	**	-	-	***	*	-		-	**	-
H20W5	****	-	-	****	*	**	-	-	*	-	-	-	*	**	-	**	-	-	-	*	-		****	**	**	****
H10W100	****	****	****	****	****	****	-	-	*	-	-	-	*	***	*	****	-	-	-	**		-	****	*	*	****
H10W50	****	****	****	****	****	****	-	-	-	*	***	-	-	-	-	-	-	*	*		-	*	****	*	*	****
H10W20	****	-	-	****	**	**	-	-	*	-	-	-	*	**	*	***	-	-		-	-	-	****	**	*	****
H10W10	****	-	-	****	*	**	-	-	*	-	-	-	*	**	*	**	-		-	-	-	*	**	-	-	**
H10W5	****	****	****	****	****	****	-	-	-	-	*	-	-	-	-	-		-	-	-	-	-	***	*	*	****
H5W100	****	****	****	****	****	****	**	*	-	***	****	-	-	-	-		****	**	****	****	****	****	-	*	****	-
H5W50	****	****	****	****	****	****	-	-	-	*	***	-	-	-		-	****	*	****	****	****	****	-	-	***	-
H5W20	****	****	****	****	****	****	**	-	-	**	****	-	-		-	-	****	**	****	****	****	****	-	-	***	-
H5W10	****	****	****	****	****	****	*	-	-	*	**	-		-	-	-	***	*	****	****	***	****	-	-	*	-
H5W5	****	****	***	****	****	****	-	-	-	-	-		-	-	-	-	*	-	*	-	-	**	-	-	-	-
H2.5W100	****	****	****	****	****	****	-	-	***	-		-	-	**	**	***	**	-	***	***	***	***	**	-	-	***
H2.5W50	****	****	****	****	****	****	-	-	*		-	-	*	****	***	****	*	-	**	*	*	**	***	-	-	****
H2.5W20	****	****	****	****	****	****	-	-		-	**	-	***	****	****	****	-	-	-	-	-	*	***	-	-	****
H2.5W10	****	****	****	****	****	****	-		-	-	-	-	**	***	***	****	-	-	-	-	-	*	**	-	-	****
H2.5W5	****	**	**	****	****	****		-	-	-	-	-	**	***	**	***	-	-	-	-	-	-	**	-	-	***
H0W100	****	***	***	****	-		-	-	*	-	-	-	*	***	***	****	**	-	**	**	**	**	**	-	-	****
H0W50	****	**	**	****		**	*	**	***	**	-	-	-	*	-	**	***	-	****	****	****	****	-	-	*	*
H0W20	***	****	****		-	***	**	**	****	****	**	-	-	-	-	*	****	*	****	****	****	****	-	-	**	-
H0W10	****	-		-	-	**	*	*	***	**	-	-	-	-	-	*	***	-	****	****	****	****	-	-	*	*
H0W5	****		****	****	****	-	-	-	-	-	-	-	**	****	****	****	*	-	*	*	*	**	**	-	-	****
C		****	****	****	****	****	****	****	****	****	****	**	*	*	***	**	****	****	****	****	****	****	-	****	****	****

Table S5. Statistical analysis for the minor axis length of activated keratocytes (red) and nuclei (blue) for all conditions with respect to each other. – Not significant, * p<0.05, ** p<0.01, *** p<0.001, **** p<0.0001.

H20W100	****	****	****	–	****	****	–	**	****	****	****	–	*	****	****	****	****	***	–	****	****	****	****	****	****	
H20W50	****	****	****	****	****	****	****	****	****	****	****	****	****	****	****	****	*	–	****	****	****	****	*	***		–
H20W20	****	****	****	****	****	****	****	****	****	****	****	****	****	****	****	****	–	**	****	****	****	****	–		*	*
H20W10	****	****	****	****	****	****	****	****	****	****	****	****	****	****	****	****	–	**	****	****	****	***		–	–	–
H20W5	****	****	****	****	****	****	****	****	****	****	****	****	****	****	****	****	****	****	****	****	****		–	–	–	–
H10W100	–	****	***	****	–	–	***	–	–	*	****	*	–	–	*	****	****	****	****	*		****	****	****	****	****
H10W50	*	****	–	****	***	***	*	–	–	****	****	–	–	–	****	****	****	****	****		*	**	***	****	***	****
H10W20	****	****	****	**	****	****	–	****	****	****	****	*	***	****	****	****	****	*		–	**	–	–	**	–	–
H10W10	****	****	****	****	****	****	****	****	****	****	****	****	****	****	****	****	**		–	–	**	–	–	*	–	–
H10W5	****	****	****	****	****	****	****	****	****	****	****	****	****	****	****	****		****	****	****	****	****	****	**	****	****
H5W100	****	**	****	****	****	****	****	****	****	****	**	****	****	****	**		****	**	**	**	–	****	****	****	****	****
H5W50	****	–	****	****	*	–	****	****	**	–	–	***	**	**		–	****	–	–	–	–	**	***	****	***	****
H5W20	–	****	*	****	–	–	**	–	–	**	****	–	–		–	–	****	**	**	**	–	****	****	****	****	****
H5W10	–	***	–	**	*	*	*	–	–	**	****	–		–	*	–	****	***	***	****	**	****	****	****	****	****
H5W5	*	****	–	–	**	**	–	–	*	****	****		–	–	–	–	****	**	**	**	–	****	****	****	****	****
H2.5W100	****	–	****	****	****	**	****	****	****	–		*	–	**	****	***	****	****	****	****	****	****	****	****	****	****
H2.5W50	****	*	****	****	–	–	****	****	**		–	*	–	***	****	***	****	****	****	****	****	****	****	****	****	****
H2.5W20	–	****	*	****	–	*	***	–		**	*	–	–	–	**	–	****	****	****	****	***	****	****	****	****	****
H2.5W10	*	****	–	****	***	***	*		–	–	–	–	–	–	**	–	****	****	****	****	****	****	****	****	****	****
H2.5W5	****	****	–	–	****	****		***	**	****	****	–	*	–	–	–	****	–	–	–	–	**	***	****	**	***
H0W100	****	***	****	****	–		****	****	****	****	****	****	****	****	****	****	****	****	****	****	****	****	****	****	****	****
H0W50	***	****	****	****		****	****	****	****	****	****	****	**	****	****	****	****	****	****	****	****	****	****	****	****	****
H0W20	****	****	****		****	****	*	–	–	**	**	–	–	–	**	–	****	***	****	****	***	****	****	****	****	****
H0W10	****	****		****	****	****	****	–	*	–	–	*	–	***	****	***	****	****	****	****	****	****	****	****	****	****
H0W5	****		****	****	****	**	****	****	****	****	****	****	****	****	****	****	****	****	****	****	****	****	****	****	****	****
C		****	****	***	****	****	*	*	–	****	****	–	–	–	**	–	****	****	****	****	***	****	****	****	****	****
	C	H0W5	H0W10	H0W20	H0W50	H0W100	H2.5W5	H2.5W10	H2.5W20	H2.5W50	H2.5W100	H5W5	H5W10	H5W20	H5W50	H5W100	H10W5	H10W10	H10W20	H10W50	H10W100	H20W5	H20W10	H20W20	H20W50	H20W100

Table S6. Primer sequences used for RTqPCR analysis of gene expression.

**Peptidylprolyl isomerase A (PPIA)**	For	GGCAAATGCTGGACCCAACACA
	Rev	TGCTGGTCTTGCCATTCCTGGA
**Aldehyde dehydrogenase 3 Family member A1 (ALDH3A1)**	For	CTCGTCATTGGCACCTGGAACT
	Rev	CTCGCCATGTTCTCACTCAGCT
**Lumican (LUM)**	For	AACATACCAACTGTCAATGAAAACC
	Rev	TGCCATCCAAACGCAAATGCTTG
**Keratocan (KER)**	For	TGCTCATCTGCAGCACCTTCAC
	Rev	ATCCAGACGGAGGTAGCGAAGA
**Alpha smooth muscle actin (α-SMA)**	For	CTATGCCTCTGGACGCACAACT
	Rev	CAGATCCAGACGCATGATGGCA
**Cluster of differentiation 90 molecule (CD90)**	For	GAAGGTCCTCTACTTATCCGCC
	Rev	TGATGCCCTCACACTTGACCAG
**Vimentin (VIM)**	For	AGGCAAAGCAGGAGTCCACTGA
	Rev	ATCTGGCGTTCCAGGGACTCAT
**Cluster of differentiation 34 molecule (CD34)**	For	CCTCAGTGTCTACTGCTGGTCT
	Rev	GGAATAGCTCTGGTGGCTTGCA

Video S1. Live cell imaging of fibroblastic keratocytes on 2D protein patterns (H0W20). Imaging started 24 hours after seeding and continued for 6 days. Over time, cells adhere to both the protein pattern and the non-adhesive are in between the patterned areas.
